# Extranodal natural killer/T-cell lymphoma presenting as orbital cellulitis

**DOI:** 10.3205/oc000055

**Published:** 2017-02-06

**Authors:** Hanis Zuhaimy, Hayati Abdul Aziz, Suresh Vasudevan, Siah Hui Hui

**Affiliations:** 1 Ophthalmology Department, Hospital Sultanah Aminah Johor Bahru, Johor, Malaysia; 2 Pathology Department, Hospital Sultanah Aminah Johor Bahru, Johor, Malaysia

**Keywords:** extranodal NK/T cell lymphoma, orbital cellulitis, sinusitis

## Abstract

**Objective:** To report an aggressive case of extranodal natural killer/T-cell lymphoma (NKTCL) of the ethmoid sinus presenting as orbital cellulitis

**Method: **Case report

**Results:** A 56-year-old male presented with right eye redness, reduced vision, and periorbital swelling for 5 weeks duration associated with a two-month history of blocked nose. The visual acuity of the right eye was 6/18. The eye was proptosed with periorbital oedema and conjunctival chemosis. The pupil was mid-dilated but there was no relative afferent pupillary defect. The fundus was normal. The extraocular movements were restricted in all directions of gaze. Nasal endoscopy revealed pansinusitis that corresponded with CT scan orbit and paranasal sinuses findings. Despite treatment, he showed no clinical improvement. Ethmoidal sinus biopsies performed revealed extranodal NKTCL. Further imaging showed involvement of the right orbital contents and its adnexa with intracranial extension into the right cavernous sinus and meninges over right temporal fossa. The patient underwent chemotherapy. However he succumbed to his illness two months after the diagnosis.

**Conclusion:** Extranodal NKTCL is a great mimicker. This case demonstrated how an acute initial presentation of extranodal NKTCL can present as orbital cellulitis with pansinusitis.

## Introduction

Extranodal natural killer T-cell lymphoma (NKTCL) is a rare form of non-Hodgkin lymphoma (NHL). It is highly invasive with an extremely aggressive course. It is commonly found in Asians, Mexicans and South Americans [1]. Majority of cases present in the nasal, nasopharynx and paranasal area. The diagnosis of extranodal NKTCL is often delayed and difficult due to its wide variety of non-specific sign and symptoms. We report an aggressive case of extranodal NKTCL of the ethmoid sinuses that presented as orbital cellulitis.

## Case description

A 56-year-old Malay male presented with progressive right eye periorbital swelling, eye redness and blurring of vision for 5 weeks duration. His symptoms were preceded by a 2-month history of persistent nose block. 

**Figure 1:**
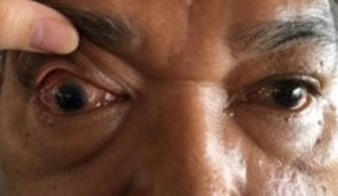
Conjunctival chemosis and proptosis of the right eye

On examination, the right eye was proptosed. The upper and lower eyelids were swollen and erythematous with conjunctival chemosis (Figure 1 (Fig. 1)). His visual acuity was 6/18 and 6/9 on the right and left eye respectively. The right pupil was mid-dilated but there was no relative afferent pupillary defect. The intraocular pressure of the right eye was 28 mmHg and the extraocular movements were restricted in all directions of gaze. The anterior segment and funduscopy examination were unremarkable. He was febrile with temperature of 38.5°C. Cranial nerves were normal and there were no palpable cervical, inguinal or axillary lymph nodes.

**Figure 2:**
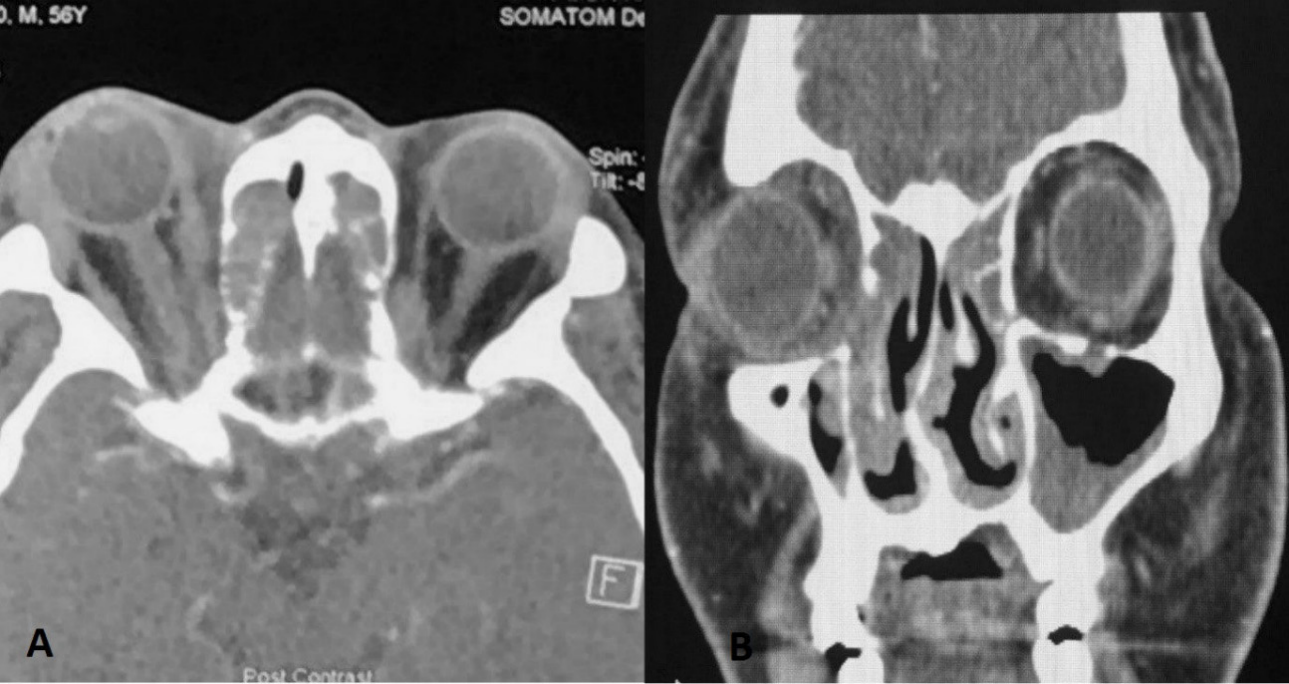
CT scan imaging of the orbit and paranasal sinuses: A) Axial view (left) demonstrated right periorbital soft tissue swelling with right extraocular muscle bulkier compared to left side. There was enlargement of the right lacrimal gland. Soft tissue density was seen within ethmoidal air cells. B) Coronal view (right) demonstrated soft tissue density in the frontal sinus, ethmoidal air cells and mucosal thickening in both the maxillary sinuses with obliteration of both osteomeatal complex. There was erosion of the right lamina papyracea.

Computed tomography (CT) scan of the orbit and paranasal sinuses (Figure 2 (Fig. 2)) revealed right periorbital soft tissue swelling and enhancement with enlargement of the right lacrimal gland. The right extraocular muscles were bulkier compared to the left with fat streakiness in the extra and intraconal spaces. There was soft tissue density within the frontal sinuses, ethmoidal air cells, and mucosal thickening in both maxillary sinuses with evidence of erosion of the right lamina papyracea. Both osteomeatal complexes were obliterated. The nasal endoscopy findings showed deviated nasal septum to the right, mucinous discharge from posterior ethmoid sinus in the right nostril with obliteration of osteomeatal complex in both nostrils suggestive of pansinusitis. No evidence of tumour or polyp was seen. 

The patient was diagnosed as orbital cellulitis secondary to pansinusitis. Intravenous amoxicillin/clavulanic acid, nasal decongestants, and anti-histamine were commenced. Culture and sensitivity of the ethmoid sinuses grew *Klebsiella pneumoniae*. However, despite a week of treatment the patient showed no clinical improvement. An urgent functional endoscopic sinus surgery (FESS) and paranasal sinus biopsies was performed as there was suspicion of an underlying sinister pathology. 

**Figure 3:**
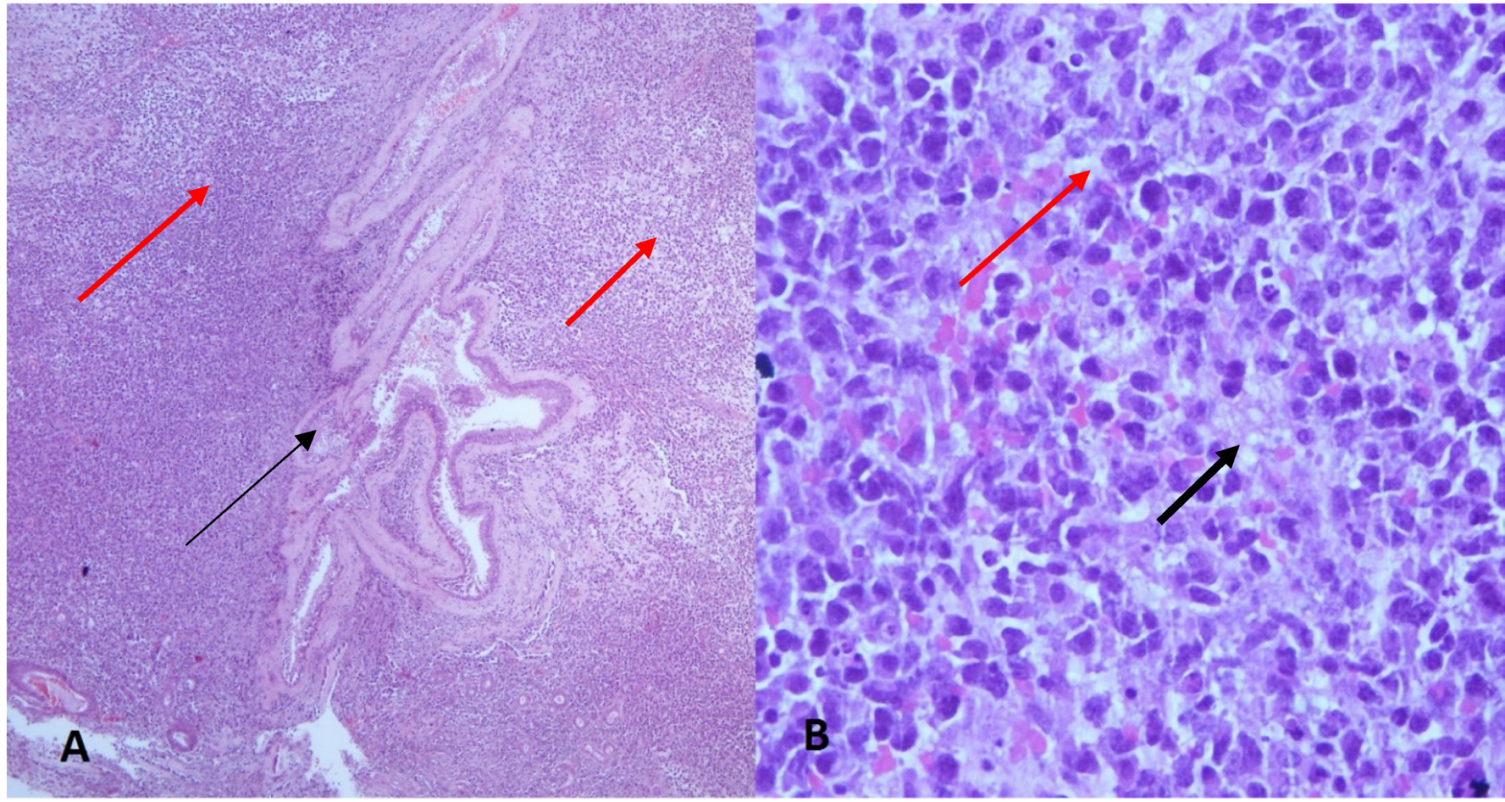
Ethmoid sinus biopsy specimen A: H&E stain (100x) showed a piece of lymphoid tissue covered by respiratory epithelium (black arrow) and the stroma was densely infiltrated by neoplastic lymphoid cells (red arrows) B: H&E stain (400x) showed dense infiltration of the stroma with neoplastic lymphoid cells. There were prominent scattered areas of necrosis and apoptotic bodies seen (black arrow).

**Figure 4:**
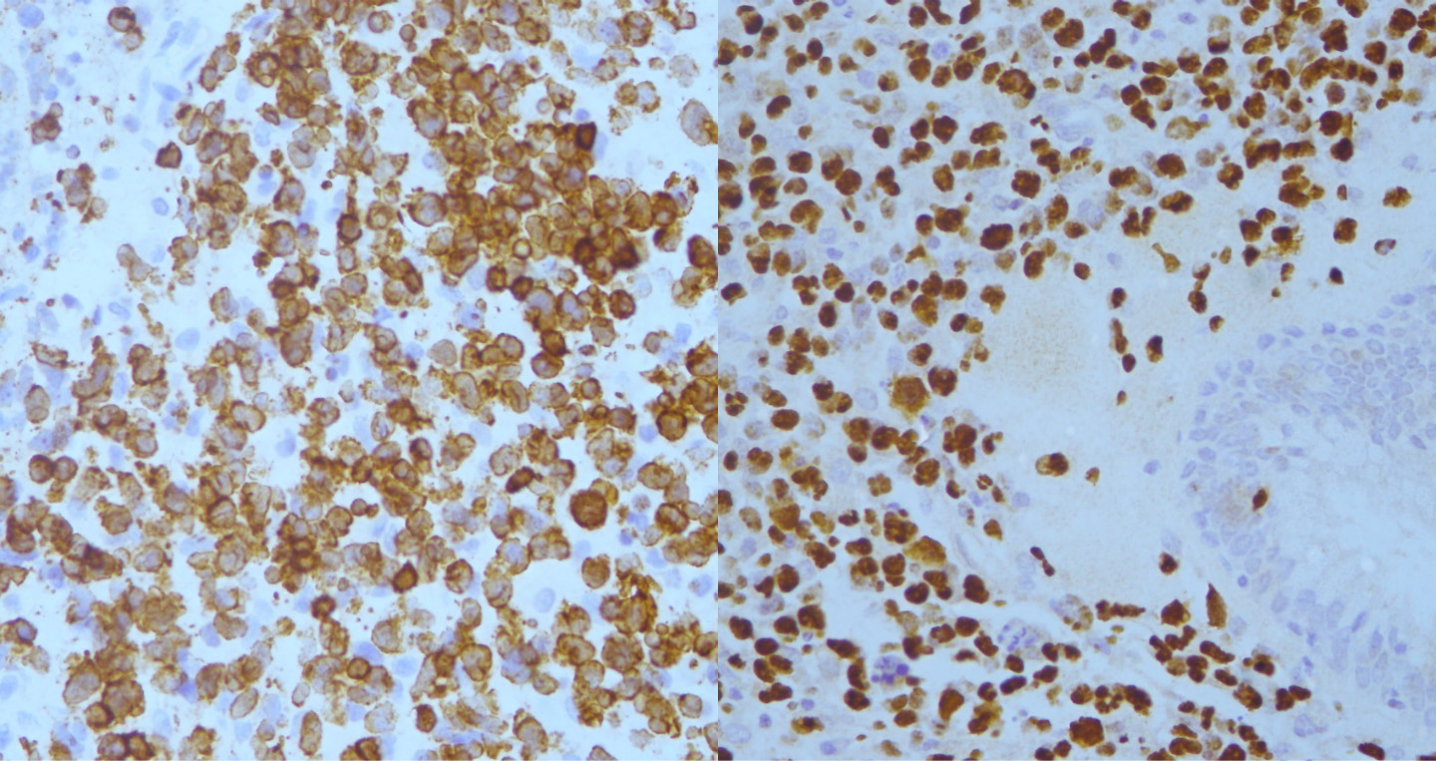
Immunohistochemical staining showed positivity for CD3 (left) and in-situ hybridization for EBV encoded RNA (right).

Histopathological specimens of the ethmoidal tissues (Figure 3 (Fig. 3)) revealed multiple fragments of fibrocollagenous tissue focally covered by respiratory epithelium containing seromucinous glands. The stroma was densely infiltrated by neoplastic lymphoid cells with areas of necrosis. Immunohistochemical staining were positive for CD3, TIA, EBER-ish and negative for CD20, CD10, tdt, cyclin-D1, CD4, CD8, CD5, CD30 and ALK (Figure 4 (Fig. 4)). The Ki-67 proliferative index was high (~60–70%). These findings were suggestive of an aggressive extranodal NK/T-cell lymphoma. 

The patient subsequently was subjected to further imaging and work out for staging purposes. Magnetic resonance imaging of brain, and orbit showed involvement of the right recti muscles, lacrimal gland, optic nerve and orbital fat with intracranial extension to the cavernous sinuses and meninges over right temporal fossa. Bone marrow biopsy showed evidence of infiltration with abnormal lymphoid cells which were positive for TIA, CD3, Ki67 and negative for CD56 and CD20. Lumbar puncture showed numerous lymphoid cells in the cerebrospinal fluid indicating central nervous system (CNS) infiltration.

The patient was diagnosed with stage IV extranodal NK/T-cell lymphoma with CNS involvement. He underwent SMILE chemotherapy (dexamethasone, methotrexate, ifosfamide, L-asparginase, etoposide). However he succumbed to his death within one month of chemotherapy, approximately 2 months after the diagnosis of extranodal NKTCL.

## Discussion

Extranodal natural killer/T-cell lymphoma (NKTCL) is a rare form of non-Hodgkin lymphoma (NHL). The tumour is derived from natural killer cells and/or cytotoxic T-lymphocytes. It accounts for only 7–10% of Non-Hodgkin lymphomas in Asia and Central America [1]. In the Asian population, it is more commonly found among the Chinese, Japanese and Koreans [2]. The disease has an unknown etiology but has been postulated to be caused by the Epstien Barr virus (EBV) infection [3].

Extranodal NKTCL is a great mimicker. Due to its wide variety of non-specific sign and symptoms the diagnosis is often delayed and difficult. It commonly presents in the nasal, nasopharynx and paranasal area in 70% of cases [3], [4]. Typically the disease presents with midline destruction of the nasal cavity, paranasal sinuses and nasopharynx giving rise to common symptoms such as nasal obstruction (87%), purulent nasal discharge (73%), and epistaxis (60%) [3]. Other possible extranasal sites include skin, breast, testis or gastrointestinal tract [4], [5]. 

Our patient first presented with orbital and concurrent sinonasal involvement. Cases of extranodal NKTCL with orbital and sinonasal involvement masquerading as orbital cellulitis with similar findings as our patient had been reported [6], [7], [8], [9], [10]. Orbital involvement is usually a result of extension or invasion from the nasal cavity and paranasal sinuses with or without bony erosion [4]. However, Woog et al. reported three out of eight cases of extranodal NKTCL had only orbital, adnexa, and/or ocular involvement [10].

Orbital cellulitis secondary to pansinusitis was diagnosed in our patient based on the clinical signs and symptoms supported by imaging and nasal endoscopy findings. In addition, ethmoid tissue C&S also revealed *Klebsiella pneumoniae* infection. Our patient showed no respond to the conventional treatment of orbital cellulitis leading to a suspicion of other underlying sinister pathology. Hence an urgent histopathological study was warranted. 

Extensive necrosis of the sinuses in pansinusitis are often misinterpreted as an infectious process rather than extranodal NKTCL superimposed with infection [7]. This was demonstrated in our patient’s CT report and nasal endoscopy finding. Due to the nature of the disease that is characterized by angioinvasion and midline destruction, biopsies for extranodal NKTCL are most of the time inconclusive. Histopathological findings often show prominent necrosis and mixture of inflammatory cells [7]. Previous case reports have highlighted the importance of adequate initial biopsies and repeated biopsies to diagnose extranodal NKTL [6], [8]. We managed to arrive at an early diagnosis in our patient due to multiple tissue biopsies taken from the ethmoid sinuses. 

Despite an early diagnosis, our patient unfortunately was found to be at an advanced stage of the disease. He was diagnosed with extranodal NKTCL stage IV with central nervous system involvement according to the Ann Arbor classification for staging of lymphoma (Table 1 (Tab. 1)). SMILE chemotherapy was commenced. However he succumbed to his illness after 2 months of diagnosis. 

Current treatment strategies for extranodal NKTCL depend on extent and stage of disease. According to the Ann Arbor classification, the disease can be classified into four stages [11]. Localised disease (stage I/II) is treated with chemotherapy and radiotherapy while disseminated disease (stage III/IV) is given combined chemotherapy. The prognosis of extranodal NKTCL described in literature varies, but generally the disease is rapidly progressive with poor prognosis and short survival times from the time of diagnosis. It has a high mortality rate and low response to chemotherapy and radiotherapy in comparison to other subtypes of neck and head lymphomas with an average survival of 12.5 months after diagnosis [7]. Seven out of eight patients with extranodal NKTCL in a cases series by Woog et al. died 5 weeks to 13 months after presentation and only one survived with no evidence of disease at 5 years after treatment [10]. Meanwhile another literature review found median survival for 26 patients with nasal-type NK/T cell lymphoma was only 7.4 months [12].

In conclusion, extranodal NKTCL is highly invasive and has an aggressive course. The prognosis is poor with a high mortality rate. This case demonstrated how an acute initial presentation of extranodal NKTCL nasal type can present as orbital cellulitis with pansinusitis. A non-responsive orbital cellulitis and sinusitis to conventional therapy should alert the ophthalmologist to the possibility of extranodal NKTCL.

## Notes

### Acknowledgment

We would like to thank our colleagues in the Department of Ophthalmology, Hospital Sultanah Aminah Johor Bahru for their support. 

### Competing interests

The authors declare that they have no competing interests.

## Figures and Tables

**Table 1:**
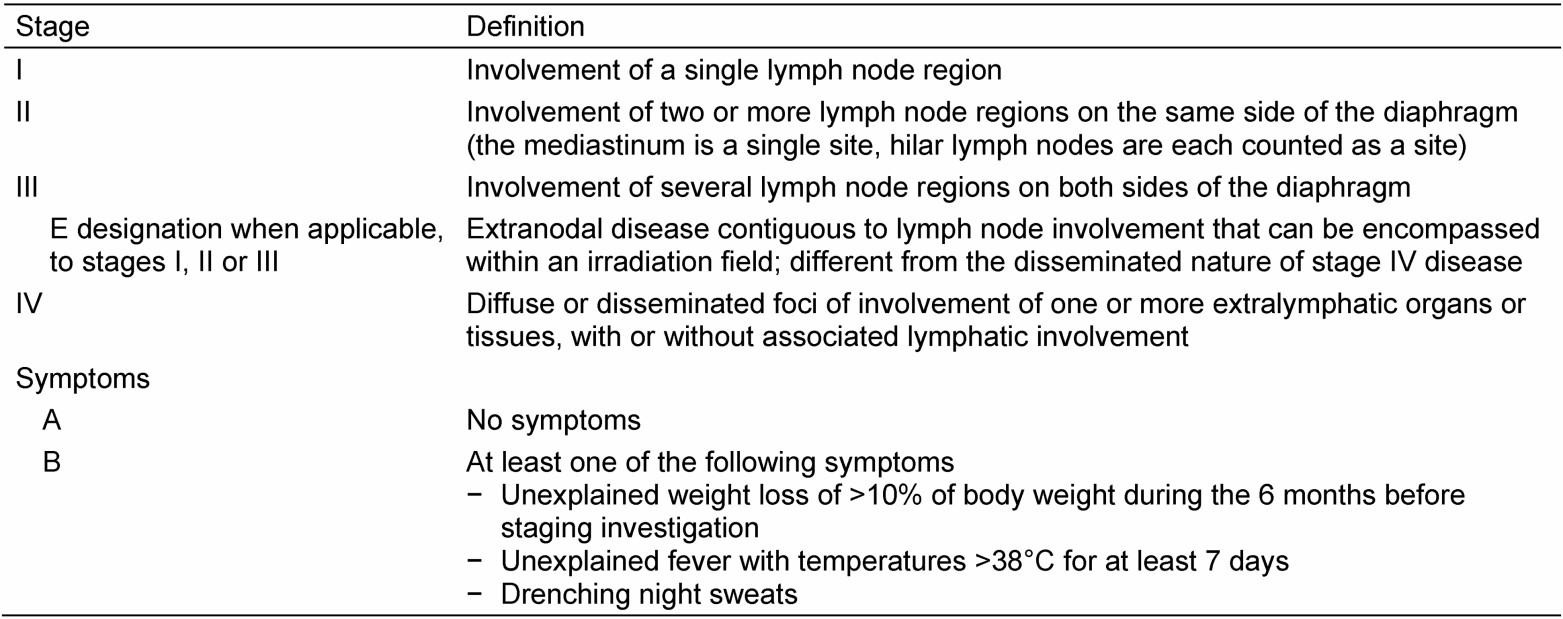
Ann Arbor staging for lymphoma
